# Designing an implementation science clinical trial to integrate hypertension and cardiovascular diseases care into existing HIV services package in Botswana (InterCARE)

**DOI:** 10.1186/s13063-024-08333-0

**Published:** 2024-07-29

**Authors:** Nabila Youssouf, Gaone Edwin Mogaetsho, Thato Moshomo, Tendani Gaolathe, Ponego Ponatshego, Mareko Ramotsababa, Onkabetse Julia Molefe-Baikai, Evelyn Dintwa, Tsaone Kiki, Amelia E. Van Pelt, Karen Steger-May, Laura M. Bogart, Shabbar Jaffar, Pooja Gala, Duolao Wang, Khumo Seipone, Kara Bennett, Kathleen Wirth Hurwitz, Kago Kebotsamang, Lisa R. Hirschhorn, Mosepele Mosepele

**Affiliations:** 1 London School of Hygiene and Tropical Medicine, London, UK; 2 Botswana Harvard Health Partnership, Gaborone, Botswana; 3 Faculty of Medicine, University of Botswana, Gaborone, Botswana; 4 Department of Medical Social Sciences, Northwestern University Feinberg School of Medicine, Chicago, IL USA; 5 Center for Biostatistics and Data Science, Washington University School of Medicine, St. Louis, MO USA; 6 RAND Corporation, Santa Monica, CA USA; 7 Charles R. Drew University of Medicine and Science, Los Angeles, CA USA; 8 University College London, London, UK; 9 Department of Medicine, NYU Langone Grossman School of Medicine, New York, NY USA; 10 Liverpool School of Tropical Medicine, Liverpool, UK; 11 African Comprehensive HIV/AIDS Partnership (ACHAP), Gaborone, Botswana; 12 Target RWE, Durham, NC USA; 13 Target RWE Company, Durham, NC USA; 14 Department of Statistics, Faculty of Social Sciences, University of Botswana, Gaborone, Botswana

**Keywords:** HIV, Hypertension, Cardiovascular disease, Botswana, Care integration, Implementation research, Treatment partners, Electronic health record

## Abstract

**Background:**

Despite success in HIV treatment, diagnosis and management of hypertension (HTN) and cardiovascular disease (CVD) remains suboptimal among people living with HIV (PLWH) in Botswana, with an overall HTN control of only 19% compared to 98% HIV viral suppressed. These gaps persist despite CVD primary care national guidelines and availability of free healthcare including antihypertensive medications. Our study aims to develop and test strategies to close the HTN care gap in PLWH, through integration into HIV care, leveraging the successful national HIV care and treatment program and strategies.

**Methods:**

The InterCARE trial is a cluster randomized controlled hybrid type 2 effectiveness-implementation trial at 14 sites designed to enroll 4652 adults living with HIV and HTN plus up to 2326 treatment partners. Primary outcomes included effectiveness (HTN control) and implementation outcomes using the Reach Effectiveness Adoption Implementation and Maintenance framework, with explanatory mixed methods used to understand variability in outcomes.

InterCARE trial’s main strategies include healthcare worker HTN and CVD care training plus long-term practice facilitation, electronic health record (EHR) documentation of key indicators and use of reminders, and use of treatment partners to provide social support to people living with HIV and HTN. InterCARE started with formative research to identify contextual factors influencing care gaps using the Consolidated Framework for Implementation Research. Results were used to adapt initial and develop additional implementation strategies to address barriers and leverage facilitators. The package was pilot tested in two clinics, with findings used to further adapt or add strategies for the clinical trial.

**Discussion:**

If successful, the InterCARE model can be scaled up to HIV clinics nationwide to improve diagnosis, management, and support in Botswana. The trial will provide insights for scale-up of HTN integration into HIV care in the region.

**Trial registration:**

ClinicalTrials.gov reference NCT05414526. Registered 18 May 2022, https://clinicaltrials.gov/study/NCT05414526?term=NCT05414526.&rank=1.

## Administrative information

**Table:** 

Title {1}	Designing an implementation science clinical trial to Integrate hypertension and cardiovascular diseases care into existing HIV services package in Botswana (InterCARE)
Trial registration {2a and 2b}	Reference NCT05414526. ClinicalTrials.gov.
Protocol version {3}	27 November 2023. V4.
Funding {4}	This trial is funded by the United States of America National Institute of Health (NIH) via the National Heart, Lung and Blood Institute (NHLBI): Federal Award Identification Number (FAIN) UG3HL154499.
Author details {5a}	London School of Hygiene and Tropical Medicine, United Kingdom1 Botswana Harvard Health Partnership, Botswana2 Faculty of Medicine, University of Botswana, Botswana3 Department of Medical Social Sciences, Northwestern University Feinberg School of Medicine, Chicago, Illinois, United States4 Center for Biostatistics and Data Science, Washington University School of Medicine, St. Louis, MO/United States5 RAND Corporation, Santa Monica CA, USA6 Charles R. Drew University of Medicine and Science, Los Angeles, CA, USA7 University College London, UK8 Department of Medicine, NYU Langone Grossman School of Medicine, New York, NY, USA9 Liverpool School of Tropical Medicine, UK10 African Comprehensive HIV/AIDS Partnership (ACHAP), Botswana11 Bennett Statistical Consulting, Inc, Ballston Lake, NY, USA12 NoviSci, Inc., a Target RWE Company, Durham, North Carolina, USA13 Department of Statistics, Faculty of Social Sciences, University of Botswana14
Name and contact information for the trial sponsor {5b}	This trial is funded by the United States of America National Institute of Health (NIH) via the National Heart, Lung and Blood Institute (NHLBI): https://clinicaltrials.gov/study/NCT05414526?term=NCT05414526.&rank=1
Role of sponsor {5c}	The funders had a role in the study design but have no role in data collection, nor future analysis, interpretation, or writing of the trial manuscript(s).

## World Health Organization Trial Registration Data Set {2b}

**Table:** 

Data category	Information
Primary registry and trial identifying number	ClinicalTrials.gov NCT05414526
Date of registration in primary registry	18 May 2022
Secondary identifying numbers	InterCARE project
Source(s) of monetary or material support	United States of America National Institute of Health (NIH) via the National Heart, Lung and Blood Institute (NHLBI):
Primary sponsor	United States of America National Institute of Health (NIH) via the National Heart, Lung and Blood Institute (NHLBI):
Secondary sponsor(s)	N/A
Contact for public queries	Mosepele Mosepele, MD. mosepele.mosepele@gmail.com
Contact for scientific queries	Mosepele Mosepele, MD. mosepele.mosepele@gmail.com
Public title	Integrating Hypertension and Cardiovascular Diseases Care Into Existing HIV Services Package in Botswana (InterCARE)
Scientific title	Designing an implementation science clinical trial to Integrate hypertension and cardiovascular diseases care into existing HIV services package in Botswana (InterCARE)
Countries of recruitment	Botswana
Health condition(s) or problem(s) studied	Hypertension, Cardiovascular disease, HIV
ntervention(s)	Active Comparator: Integrating hypertension and cardiovascular diseases care (InterCARE). Placebo comparator: standard of care/routine care
Key inclusion and exclusion criteria	Inclusion 1) aged between 20 and 75 years old 2) have documented HIV infection and on ART 3) confirmed diagnosis of HTN or elevated systolic blood pressure or diastolic blood pression 4) registered client at the selected clinics All sexes eligible, doesn’t’ accept healthy volunteers Exclusion Documented as having a diagnosis of dementia in clinic records, or unable or unwilling to provide informed consent.
Study type	Mixed-methods, cluster randomized control type II hybrid effectiveness-implementation trial
Date of first enrolment	January 2023
Target sample size	4,652 PLWH with HTN across the intervention and control sites. Up to 2,326 treatments partners
Recruitment status	Follow up
Primary outcome(s)	Effectiveness (proportion of PLHIV with diagnosed hypertension receiving anti-hypertensive medication with controlled blood pressure) Implementation (Proportion of clinic encounters in EHR where anti-hypertensive medications are prescribed if indicated)
Key secondary outcomes	Reach (proportion of PLWHIV with hypertension who are aware of their hypertensive status) Adoption (proportion of PLWHIV with HTN prescribed anti-hypertensive medications in the EHR) Implementation fidelity (audit of intervention implementation as designed) Implementation feasibility (ability to implement integrated HIV/HTN care, HIV/HTN/CVD care etc.) Implementation acceptability (patient and provider survey and interviews) of the implementation strategy. Maintenance (provider and patient perceptions of ability to maintain plus change in blood pressure control from 12-24 months of the trial).

## Introduction

### Background and rationale {6a}

Globally, people living with HIV (PLWH) have longer life expectancy due to improvement in access to HIV care, including antiretroviral therapies (ART). As lifespan has increase, this population is aging, with growing rates of cardiovascular diseases (CVDs) and other age-related morbidities. An estimated 24% of PLWH have hypertension (HTN), making it one of the most prevalent modifiable risk factors for CVD in this population [1]. HTN occurs among younger individuals compared to the general population, with a median age of about 38 years in most cohorts of PLWH in Sub-Saharan Africa [2]. Gaps in diagnosis and treatment exist, with rates of treatment and control are well below 50% among PLWH with HTN in Botswana [3]. This gap in HTN care extends to other countries such as Uganda, where 88% of PLWH and HTN were initiated on blood pressure treatment, but only 24.3% achieved blood pressure control [4]. These lower rates of HTN treatment and control are in contrast to the success of Botswana in surpassing the 2022 Joint United Nations Program on HIV/AIDS (UNAIDS) targets of 95% diagnosis, treatment, and viral suppression [3]. Therefore, innovative strategies are required to leverage the successes of the HIV program to address NCD challenges such as hypertension among PLWH and HTN.

One in 4 adult PLWH in Botswana have HTN, making it one of the leading CVD risk factors in this population [5, 6]. Botswana recognized the increased burden of non-communicable diseases (NCDs), reflected in the national Multisectoral Strategy for the Prevention and Control of NCDs published by the Ministry of Health (MOH) [7]. The strategy included national guidelines for HTN diagnosis and treatment and free universal healthcare including HTN medications. The strategy also included recommendations for integrating HTN into HIV care, leveraging the existing HIV care program plus electronic documentation of HTN-related national indicators. However, little is known about the specific strategies required to achieve this goal in Botswana.

### Objectives {7}

The hypotheses of this study are as follows:

- Integrating hypertension care within an existing HIV care platform is feasible, acceptable, and associated with early improvement in the hypertension cascade of care but will require substantial change in patient and provider attitudes, knowledge, and clinic operations.
- The study’s proposed multi-component intervention will largely be successful, as measured by the Reach, Effectiveness, Adoption- Implementation, Maintenance (RE-AIM) framework.

### Trial design {8}

The INTEgrating hypeRtension and cardiovascular diseases CARE into existing HIV services package in Botswana** (**InterCARE) trial is a mixed-methods, cluster randomized control type II hybrid effectiveness-implementation trial in fourteen HIV clinics in Botswana.

## Methods: participants, interventions, and outcomes

### Study setting {9}

This study will be conducted within the ~ 497 Botswana national HIV clinic/health post system. The nationwide HIV clinics serves > 320,000 PLWH and utilizes both paper-based and electronic health records. At each HIV clinic, staffing typically includes 0–2 medical officers [sometimes resident and other times on rotation or available telephonically from other health facilities], 1–4 nurses, pharmacy technician (sometimes), variable number of nursing assistants, phlebotomist, and a part-time social worker/health welfare educator. We will choose 7 pairs of clinics from 30 communities which participated in the Botswana Combination Prevention Project (BCPP) [8]. We will obtain updated data on all 30 BCPP communities and match HIV clinics on number and type of staffing and number of patients total by using the technique of calculating Euclidean distances between all possible pairs to determine the best (closest) two matches. We may also use other factors for matching, such as extent of existing pharmacy, laboratory, and rural/urban location, if we find that factors related to blood pressure control may vary across clinics.

### Eligibility criteria {10}

Patients are eligible if they (1) are aged between 20 and 75 years old, (2) have documented HIV infection and are on ART, (3) have a confirmed diagnosis of HTN or elevated systolic blood pressure (SBP) ≥ 140 or diastolic blood pression (DBP) ≥ 90 mmHg (or SBP ≥ 130 or DBP 80 mmHg if they have diabetes mellitus or chronic kidney disease), and (4) are a registered client at the selected clinics. PLWH are not eligible if they are documented as having a diagnosis of dementia in clinic records or if they are unable or unwilling to provide informed consent. PLWH and HTN will be selected for enrolment using a purposive random sampling method by trained research assistants at each site.

### Who will take informed consent? {26a}

Trained research assistants will take written informed consent. The informed consent process will occur in the language most comfortable for participants (usually in Setswana). Permission/assent will be documented by having the participant sign the permission/assent form or make a mark if the participant is illiterate; the latter will be witnessed by a third party. A copy of the informed consent/assent form will be offered to the participant. Staff will ensure that the participant takes a copy of a study contact card that contains study information and telephone numbers if the participant does not wish to take a copy of the permission/assent form.

### Additional consent provisions for collection and use of participant data and biological specimens {26b}

Not applicable, all our consent provisions are stated in the relevant section and we will not be collecting biological specimens.

## Interventions

### Explanation for the choice of comparators {6b}

(i) *Active comparator/treatment arm*: Our treatment arm will be composed of clinics with interventions for integrating hypertension and cardiovascular diseases care (InterCARE), namely, provider training on HTN/CVD, the use of adapted electronic health record (EHR)—especially electronic reminders, and use of treatment partners for PLWHIV with hypertension.
(ii) *Placebo comparator/control arm*: Given our set of interventions as the treatment arm, the control arm will be composed of clinics without integrated care of HIV and hypertension. Care in these clinics is as usual, with no EHR modifications and no organized-updated training of health care workers on hypertension management. Thus, their training would have been at nursing/medical school or at some point in the past. Enrolled participants also do not have designated treatment partners. With this placebo comparator, hypertension is managed by a provider using any guidelines of their choosing.

### Intervention description {11a}

The InterCARE package consists of three strategies adapted from the successful Botswana HIV program: provider training, use of an EHR, and treatment partners.

- *Electronic health records (EHR)*: at the beginning of the national ART program, Botswana developed a local EHR, patient information management system (PIMS) that has been used to document outcomes of the national ART program [9], and optimized for an HIV prevention trial [3]. However, this EHR system has not been used for other chronic diseases in Botswana. The use of clinical decision support in EHR is generally associated with improvement in quality of care [10], but data are mixed for CVD risk factors monitoring. This strategy can address barriers in knowledge and adoption of CVD risk assessment and HTN management and monitoring of national strategy key NCD care indicators.
- *Treatment partners*: many national HIV programs including in Botswana have utilized community members as treatment partners for PLWH [11–15]. In Botswana, the use of treatment partners has been associated with improved HIV medication adherence and viral suppression [11]. As with other chronic lifestyle-related medical conditions, family or presence of close other persons is recommended as part of an evidence-based strategy to assist those with hypertension to care for themselves, provide social support, promote adherence to lifestyle changes/medications, etc. [11]. This American Heart Association (AHA) evidence-based strategy for social support has not been adapted for PLWH with HTN in Botswana [16]. InterCARE participants will be given guidance on how to choose an appropriate treatment partner for their HTN support. Treatment partners will attend training along with the participant on how to provide social, emotional, and adherence support.
- *HTN/CVD training*: the highly successful KITSO training program (healthcare curriculum for HIV care in Botswana) is in use at more than 400 peri-urban and rural HIV clinics in Botswana but has never been leveraged to enhance NCD care for PLWH in Botswana. Building on the model, a dedicated healthcare provider curriculum and certification on hypertension control and CVD care among PLWH was developed for use in InterCARE, reflecting national and international guidelines for HTN management. The training will be provided at the start of InterCARE and again annually. Application and ongoing learning will be supported through practice facilitation by research nurses during regularly scheduled visits and monthly data audit and feedback.

These strategies from the HIV program were refined through key partner engagement including patient groups, healthcare professionals, Ministry of Health (MoH) representatives (Information Technology, HIV program, NCD program), implementation science experts, and biostatisticians. After adaptation, these implementation strategies were piloted over a 12-month period in two clinics to test feasibility, adoption, acceptability, and fidelity. After the pilot completion, the same partner groups were reconvened to provide input for further adaptation as needed for use in the InterCARE trial.

### Criteria for discontinuing or modifying allocated interventions {11b}

Criteria for discontinuing participant follow-up include withdrawal of consent, serious harm resulting from the trial, contamination/cross-over from one arm of the study to the other, and transfer from study sites to non-study sites. Criteria for modifying allocated interventions include significant changes in study site processes, for instance a change in EHR from PIMS (system in place at current trial sites) to IPMS (system available at other health centers in Botswana, but not yet part of the InterCARE sites) by intervention sites or from IPMS to PIMS by control sites.

### Strategies to improve adherence to interventions {11c}

We believe that our EHR modifications to include prompts for recording anthropometry, cardiovascular disease risk factors, and blood pressure, among others, facilitate adherence to our intervention protocols. Furthermore, we have planned 6-monthly refresher trainings and retraining in sites where the staff turnover exceeds 50%. Pairing of patients with treatment partners will facilitate communication between them to support medication and lifestyle adherence as well as attendance of clinic appointments. We will also have patient/treatment partner booster sessions conducted via telephone: for patients with controlled BP, booster calls will take place about 3 months after initial clinic training session; for patients with uncontrolled BP, booster calls will take place on a monthly basis after initial clinic training, until BP is controlled.

Our DCF includes a question on self-reported medication adherence, we however do not directly monitor drug levels for adherence.

### Relevant concomitant care permitted or prohibited during the trial {11d}

Patients in both the intervention and control arms will continue to receive concomitant care for all the comorbidities, and we do not prohibit them from receiving any interventions during the trial.

### Provisions for post-trial care {30}

Participants with uncontrolled blood will be referred to their primary health care workers for optimization of blood pressure. Adverse events will be reported as per protocol and Botswana Medicines Regulatory Authority (BOMRA). Given the nature of our implementation science trial, we do not anticipate frequent nor serious harm as a direct effect of our study. We do not offer any compensation to those who could suffer harm from being on treatment for hypertension treatment.

### Outcomes {12}

The primary and secondary outcomes are based on the RE-AIM framework with effectiveness and implementation being the co-primary outcomes.

Primary and secondary effectiveness and implementation outcomes are fully described in Table 1. Exploratory secondary outcomes will include the extent to which participants are screened for other cardiovascular risk factors, counseled, and educated and documented in the EHR.

**Table 1:** Effectiveness and implementation outcomes for InterCARE

Co-primary outcomes		
Effectiveness	Proportion of PLWH with diagnosed hypertension receiving anti-hypertensive medication with controlled blood pressure	EHR
Implementation	Proportion of clinic encounters in EHR where anti-hypertensive medications are prescribed if indicated	EHR
Secondary outcomes		Sources
Reach	· Proportion of eligible PLWH who agreed to be enrolled into InterCARE among those who met inclusion criteria · Proportion of PLWH who are aware of their hypertension status	· Modified EHR, DCF
Effectiveness	· Absolute difference in systolic and diastolic blood pressure between baseline, 12-month, and 24-month visits · The proportion of participants who scored > 70% on the heart disease fact questionnaire (HDFQ) · Proportion of PLWHIV with hypertension prescribed anti-hypertensive medications	· Modified EHR, DCF
Adoption	· Proportion of PLWH and HTN with 10-year CVD risk documented in EHR (E-CVDRF eval) · Proportion with BP documented in EHR · Proportion of those screened for other CVD risk factors using EHR · Proportion of those who received CVD risk factor counseling on anti-HTN medication, healthy diet, and appropriate physical activity levels · Proportion of PLWHIV with HTN enrolled in InterCARE receiving HTN and HIV care in the same clinic visit · Proportion of PLWHIV with HTN with BP control not on medication	· Modified EHR, DCF
Fidelity	· Proportion prescribed guideline concordant BP medication (as per Botswana 2016 Primary Care Guideline [17]) and/or major publication on management of BP among black Africans [18] · Proportion of treatment partners trained per protocol · Proportion of treatment partners providing support with the same frequency as expected · Proportion of intervention clinics that implemented the three components of InterCARE	· EHR, DCF, electronic trial logs
Feasibility	· Implementation of integrated HIV/hypertension care	· EHR, facility visit tracker, monitoring reports
Acceptability	· Acceptability of the implementation strategy · Patients, treatment partners, and provider’s attitudes towards InterCARE	· Surveys/key partners interviews
Maintenance	· Proportion of PLWH with HTN enrolled in InterCARE with BP control, who maintain BP control in next study visit	· EHR, DCF

*EHR* electronic health record, *DCF* data collection form, *PLWH*, people living with HIV, *E-CVDRF eval*, electronic-cardiovascular disease risk factor evaluation

**Fig. 1:**
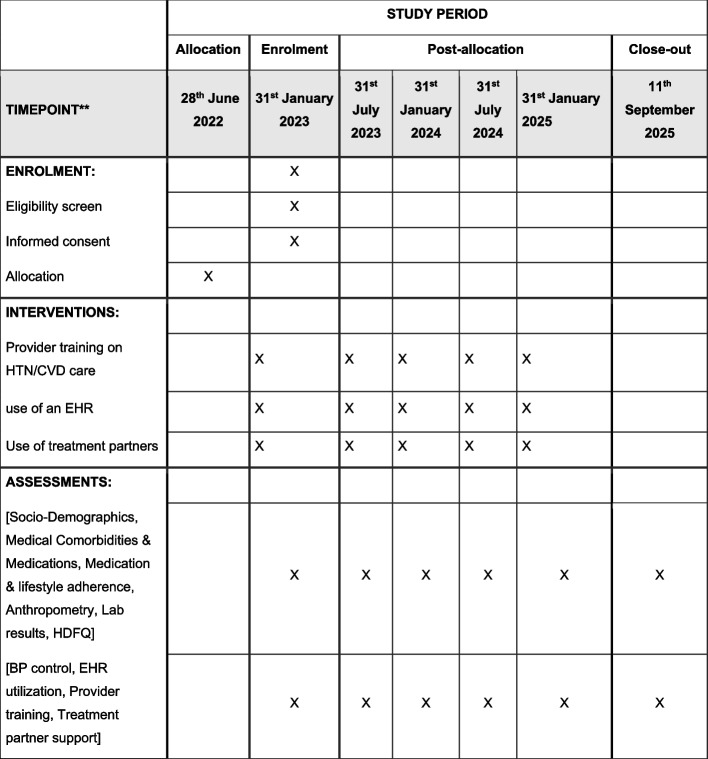
Participant timeline {13} (SPIRIT Fig. [19]): InterCARE schedule of enrolment, interventions, and assessments

See the participant timeline in Fig. 1.

### Sample size {14}

The InterCARE trial aims to recruit a total of 4652 PLWH with HTN across the intervention and control sites and up to 2326 treatments partners. Assuming unequal numbers of randomized controlled trial (RCT) enrollees per clinic, a within-clinic correlation of 0.055 [20], 10% refusal rate, 10% loss to follow-up at 12 months, a two-sided 95% confidence interval, and independent co-primary outcomes, we will need to enroll approximately 333 PLWH with hypertension on average per clinic from 14 clinics (Table 2). The number of participants to be enrolled per clinic assumes a 25% prevalence of hypertension [20] and will vary based on clinic volume. This sample size will provide > 90% power to detect achievement of intervention arm targets for all quantitative outcomes assessed within the RE-AIM framework. Of note, for all quantitative endpoint, we will have adequate power to detect smaller, yet still clinically relevant, intervention effect sizes.

**Table 2:** Power calculations based on selected RE-AIM measures

	Proportion at study end				
Endpoint	Standard of care	Intervention targets	Absolute difference	Power to reach intervention targets	Minimum detectable difference with 90% power
**Reach**					
% PLWH w/HTN aware of HTN diagnosis	46%	80%	34%	> 99%	23%
% PLWH w/diagnosed HTN receiving anti-HTN medication	42%	80%	38%	> 99%	23%
**Effectiveness**					
% PLWH w/diagnosed HTN receiving anti-HTN medication with controlled BP	19%	64%	45%	> 99%	21%
**Adoption**					
% PLWH w/HTN prescribed anti-hypertensive medications	57%	80%	23%	93%	22%
% PLWH w/HTN with waist and hip circumference recorded in the EHR	0%	50%	50%	> 99%	10%
% PLWH w/HTN with 10-year CVD risk calculated in the EHR	2%	20%	18%	98%	13%

All power calculations were conducted using formulae appropriate for a pair-matched cluster randomized trial [21].

### Recruitment {15}

Strategies for achieving adequate participant enrolment to reach target sample size is to enroll sites in pairs, i.e., one controlled site will be enrolled with one intervention site simultaneously. Before enrolment, community/stakeholder engagement followed by pre-implementation surveys will be done for each site. Training of providers on protocol and EHR changes as well as hypertension management will be done at intervention sites. Participants will be mainly enrolled during their IDCC visit (HIV-related clinic visit). To scale up enrolment, participants will also be enrolled when they came for other HIV-related services at other departments such as laboratory and pharmacy.

## Assignment of interventions: allocation

### Sequence generation {16a}

Using the coin flip randomization method, 14 clinics will be randomized to a trial arm (*n* = 7 intervention, *n* = 7 control). Since patients are registered at one clinic only (and do not tend to visit more than one clinic), participants cross-over between intervention and control clinics is unlikely; hence, we do not expect contamination to become an issue.

### Concealment mechanism {16b}

IRB approval was first granted centrally for all potentially eligible sites. Then, facility assessment readiness and willingness to participate were done. Once a rolling list of 14 sites had been reached, a study launch site was done systematically as pairs (control and associated intervention sites). Ultimately, our approach allowed for allocation concealment until randomization and study activation team was ready to activate the site.

### Implementation {16c}

Our research assistants will enroll participants who will then be exposed to the implementation strategies by healthcare providers at their HIV clinic.

## Assignment of interventions: blinding

### Who will be blinded {17a}

There was no blinding, as this is not feasible because our trial is focused on strategies to improve HTN/CVD care in the intervention sites; thus, both providers and participants ought to be aware. It was also difficult to blind our analysts as our biostatisticians work closely with the clinical operations team to track and document variability across sites, and recommend continuous adaptations, and gain understanding of why interim site data is changing.

### Procedure for unblinding if needed {17b}

This is not applicable; there was no blinding.

## Data collection and management

### Plans for assessment and collection of outcomes {18a}

#### PLWH and HTN

Following informed consent, study data from participating PLWH will be collected at baseline and then at study visits at months 6, 12, 18, and 24 for the PLWH and their treatment partners. Socio-demographics, co-morbidities, medication intake, cardiovascular health data including a blood pressure assessment, and treatment partner sessions will be collected in the study electronic database.

#### Treatment partners

A survey will be adapted from previous work on HIV treatment partners [16] to measure delivery of support to PLWH and HTN including frequency, type, and other activities. The survey will be done monthly until the BP of the patient is controlled and then every 3 months.

#### Key partners

Key partner surveys and interviews will be conducted, 1–2 months pre-implementation and then between months 55 and 60 (post-implementation). The surveys and interviews will be designed using the Consolidated Framework for Implementation Research (CFIR) 1.0 [22, 23] framework to identify potential barriers to and facilitators of implementation along the 5 CFIR domains: (1) characteristics of individuals—PLWH, health care providers, and treatment partners, (2) process of implementation of InterCARE strategy package, (3) outer setting which includes household, social network, and community, (4) inner setting (the HIV clinic), and (5) intervention (HTN management) characteristics. The surveys will be administered in English, and interview guides are translated to Setswana (local language) so that the interviews could be done in English or Setswana as needed. All key partner surveys and interviews will be face-to-face (or by phone where meeting in person is difficult). Interviews sessions will be recorded using a digital audio recording device and transcribed.

### Plans to promote participant retention and complete follow-up {18b}

We will obtain patients’ and their treatment partners’ contact information (mobile phone number, addresses) and the contact information of someone who might know their whereabouts; we will also ask permission to track them through the clinic (e.g., to find them at appointments). We will use the EHR to create a list of patients with hypertension for tracking, to ensure they attain blood pressure control (just like we have done for patients experiencing virologic failure). Both the patient and their treatment partners will be called and reminded to attend scheduled study-related sessions and treatment partner sessions.

### Data management {19}

Collected data will be managed using the Research Electronic Data Capture (REDCap) tool hosted at the University of Botswana. REDCap [24, 25] is a secure, web-based software platform designed to support data capture for research studies, providing (1) an intuitive interface for validated data capture, (2) audit trails for tracking data manipulation and export procedures, (3) automated export procedures for seamless data downloads to common statistical packages, and (4) procedures for data integration and interoperability with external sources.

### Confidentiality {27}

Confidentiality will be maintained from consent to data destruction, covering participants, their data, and study records. Identifiable data will not be shared without written consent, except for monitoring by authorized entities like IRBs, sponsors, regulators, approved monitors, or the Botswana Ministry of Health.

### Plans for collection, laboratory evaluation, and storage of biological specimens for genetic or molecular analysis in this trial/future use {33}

This is not applicable; we will not collect any biological specimens.

## Statistical methods

### Statistical methods for primary and secondary outcomes {20a}

Primary analyses will be based on the intention-to-treat principle supported by per protocol analysis. The primary effectiveness outcome will be analyzed using a generalized linear mixed model (GLMM). The model will have a binomial distribution and log link function with the treatment (treatment vs control) as fixed effect, clusters, and pairs as random effects. From GLMM, the risk ratio with 95% confidence intervals (CI) will be calculated.

In addition, adjusted GLMM model analysis will be performed with covariates measured at baseline being added into the above GLMM model. The following variables will be considered in the covariate adjusted analysis: age, sex, education, level of income, waist to hip ratios (or body mass index (BMI)), physical activity (estimated number of minutes walked per week), smoking, and alcohol consumption.

Analysis of secondary binary outcomes will be analyzed similarly as the analysis of primary outcome using GLMM models. Analysis of secondary continuous outcomes will be performed using GLMM models with normal distribution and identity link function. The models will have the treatment as fixed effect, cluster, and pair as random effects. The mean differences with 95%CIs between treatment and control will be derived from the GLMM model.

### Interim analyses {21b}

We do not have any pre-determined interim analysis and stopping guidelines as our study is low risk, and they are not required by ethics; however, using the facility and strategy tracker, we continuously assess the extent to which our strategies are implemented as planned. If they are not implemented as planned, we work with stakeholders and participants to ensure improved fidelity or adapt such strategies to promote integration of hypertension care into the HIV service platform.

## Methods for additional analyses (e.g., subgroup analyses) {20b}

### Qualitative and mixed methods analyses

We will use directed content analysis methods using the CFIR domains [22, 23]. Two team members will independently read all transcripts to develop an initial list of themes, looking for repetitions. They will then develop a codebook listing each theme accompanied by a detailed description, inclusion/exclusion criteria, and typical examples. Using a qualitative data management program, two coders will mark areas of text corresponding to each theme. They will practice by coding randomly selected transcripts independently and reviewing together. If coder disagreement reveals ambiguity in codes, we will add examples. Training will continue until coders consistently identify and mark each theme. Next, both coders will independently code a randomly selected 15% of transcripts to assess coder consistency, evidenced by Kappas of ≥ . 80 [26]. We will examine distribution of themes, overall, and by participant type. We will share the results with each partner group for feedback.

The qualitative and survey results will then be used to help understand the quantitative results of the implementation and effectiveness outcomes using explanatory mixed methods approach [27].

## Methods in analysis to handle protocol non-adherence and any statistical methods to handle missing data {20c}

### Outcomes

Primary and secondary outcomes will be carefully collected, and missing data on the primary and secondary outcomes will not be imputed, but the missingness mechanisms will be explored and factors associated with missingness would be adjusted for in exploratory analysis.

### Covariates

Missing baseline covariates will be imputed using simple imputation methods in the covariate adjusted analysis based on the covariate distribution, should the missing values for a particular covariate be less than 5%. For a continuous variable, missing values will be imputed from random values from a normal distribution with mean and SD calculated from the available sample. For a categorical variable, missing values will be imputed from random values from a multinomial distribution with probabilities ${P}_{1},{P}_{2},\dots ,{P}_{k}$ from the sample. For a count data, missing values will be imputed from random values from a Poisson distribution with $\lambda$ from the sample. If the missing values for a covariate are > 5%, then they will be imputed using Markov chain Monte Carlo (MCMC) methods using SAS PROC MI.

### Plans to give access to the full protocol, participant-level data, and statistical code {31c}

Data recorded as part of the study will be anonymized and will be deposited in a secured archiving facility at the University of Botswana Health Records Unit and the Botswana Ministry of Health Research Ethics Committee. We will also comply with funder requirements for data sharing. Researchers may request access to the data as part of transparency and data sharing; however, this is strictly upon request and will be governed through the required legal office, with at the least a data sharing agreement in place. The agreement will also detail the requirements and process for requesting access to the full protocol, a deidentified participant-level dataset, and statistical code.

## Oversight and monitoring

### Composition of the coordinating center and trial steering committee {5d}

#### Principal investigators

The principal investigators has the overall responsibility for the design and conduct of the study and preparation of the protocol and subsequent adaptations and revisions, in collaboration with the steering committee and the data safety monitoring board (DSMB).

#### Steering committee

This group, consisting of the principal investigators, study chair, study statistician, involved subject matter experts, and national leaders (representatives of site countries). The committee consists of persons with expertise in implementation science, biostatistics, epidemiology, behavioral science, infectious diseases, and internal medicine, among others. They meet on a regular basis to assess study progress and discuss necessary interventions or protocol amendments as required. Specific responsibilities include the following:

- Agreement of the final protocol and the statistical analysis plan
- Reviewing progress of the study and, if necessary, deciding on protocol changes
- Review and approval of study publications and sub-study proposals
- Reviewing new studies that may be of relevance
- Providing reports as required by the study DSMB. The DSMB was independently appointed by the study sponsor.

#### Central coordination and study management

The study will be coordinated through University of Botswana, in collaboration as a joint partnership with ACHAP, BHP, Rand Corporation, Northwestern University, LSHTM, University College of London, and WUSTL. Regular meetings are coordinated between a primary team consisting of the principal investigators, central research coordinators, and relevant team members to assess trial progress on an ongoing basis, review recruitment rates by site, and address any potential need for site visits or direct intervention. Specific responsibilities include the following:

- Study planning and organization of steering committee meetings
- Ensuring necessary regulatory and ethics committee approvals
- Development of standard operating procedures and computer systems
- Monitoring overall progress of the study
- Provision of study materials to study sites
- Monitoring and reporting safety information in line with the protocol and regulatory requirements
- Dealing with technical, medical, and administrative queries from study sites

#### Study sites

Participating facilities are represented by investigators, i.e., nurses and research assistants. Each site is led by a nurse supervisor. The investigators perform the study in accordance with Good Clinical Practice (GCP) and every new investigator provide documentation of their qualifications to the project office prior to study start. The investigator is required to ensure compliance with respect to the visit schedule and procedures required by the protocol. The investigator agrees to provide all information requested in the case report forms in an accurate and timely manner according to instructions provided. Random or for cause monitoring visits will be done by project representatives. National leaders and principal investigators are responsible for:

- Obtaining all relevant local permissions (assisted by the coordinating centers)
- All trial activities at the local site, including appropriate training and supervision for clinical staff
- Conducting trial procedures at the local site in line with all relevant local policies and procedures
- Dealing with enquiries from participants and others

### Composition of the data monitoring committee, its role and reporting structure {21a}

An independent DSMB reviews the accumulating study data on yearly basis (the frequency of review can be adjusted at their discretion based on emerging data) and makes recommendations to the study leadership about the conduct of the trial, integrity of the data, and trial discontinuation to ensure the overall safety of participants. The DSMB is provided with summaries of safety (all reportable AEs), efficacy (deaths and hospitalization), and administrative (including accrual, retention, compliance with study requirements) data.

### Adverse event reporting and harms {22}

While this protocol does not involve the administration of any study products, the study team will report in real-time, to IRBs and to the NHLBI applicable policy (https://www.nhlbi.nih.gov/grants-and-training/policies-and-guidelines/nhlbi-adverse-event-and-unanticipated-problem-reporting-policy or subsequent versions), sponsors, any serious adverse events (SAEs) experienced by a participant that in the opinion of the investigator are BOTH unexpected and at least possibly related to the research procedures. Reportable events will be reported within 7 days of the study team becoming aware of the event. The AE reporting period for this study is from the time of enrolment to the end of study follow-up for a participant.

Upon consultation with the Botswana Medicines and Health Products Regulatory Agency (BoMRA) on the pharmacovigilance reporting scope of this implementation science clinical trial, any adverse drug reactions should be reported to BoMRA using its online reporting mechanism accessible from this link: https://www.bomra.co.bw/index.php/services/patient-safety-monitoring.

Reportable events will be documented by the intervention clinic staff who will then report through the BoMRA website. The study team who will then notify the IRBs/HRDC of events that meet the SAE reporting criteria. The incident will be discussed and a written action plan will be devised and implemented within 1 week of the initial report, if needed. Written documentation of all reportable events and event resolution will be retained in the study file. These reports follow a pre-defined format as per the regulatory body. For instance, please see https://www.bomra.co.bw/index.php/suspected-adverse-drug-reactions-reporting-form for BOMRA online form and associated requirements. Reporting of events to IRBs and the study sponsor will follow institutional and IRB policies.

Adverse events (AEs) will be summarized using the number of AEs and the number (%) of participants with AEs by treatment arms. Safety analyses will summarize the number of grade III/IV AEs, graded according to Division of AIDS (DAIDS) criteria; serious adverse events (SAEs); serious adverse reactions (SARs); suspected unexpected serious adverse reactions (SUSARs); and deaths occurring after randomization. We will also measure unintended effects of HTN integration on HIV such as impact on viral suppression rates among participants.

### Frequency and plans for auditing trial conduct {23}

Fidelity to our trial procedures and interventions will be conducted weekly through the research assistant checklist (strategy and facility tracker) at 0, 12, and 24 months through an observation of training of treatment partners. Additionally, the data collection forms and modified EHR records will be audited at 0, 6, 12, 18, and 24 months for assessing treatment partner support and assessing required laboratory tests and anthropometry.

### Plans for communicating important protocol amendments to relevant parties (e.g., trial participants, ethical committees) {25}

Any changes to the protocol which may impact the conduct of the study or affect patient safety as well as any changes to the study objectives, design, patient population, sample sizes, study procedures, or significant administrative aspects will necessitate a formal amendment to the protocol. Such amendment will be agreed upon by the InterCARE team and NHLBI and approved by the UB IRB, DSMB, and the Botswana MoH HRDC prior to implementation and notified to the DHMTs.

### Dissemination plans {31a}

Our findings will be disseminated through presentations, reports, and publication in peer-reviewed journals and other publications. In-country data and country-specific information will be made available to national policy-makers and organizational and implementing partners. A detailed publication and communication plan will be developed that describes the number and types of reports and manuscripts planned for the study and criteria for authorship. Formal presentations at conferences and scientific publications will follow NIH, BHP, and MOH guidelines. The study results will be released to the participating health care workers, patients, and the general medical community.

## Discussion

While Botswana consistently reports an exemplary HIV care cascade [28], its escalating NCD burden presents a new challenge disproportionally affecting PLWH. Lessons learnt from HIV systems can be used to address the NCD burden, including HTN. The InterCARE study is the first implementation science hybrid type 2 cluster randomized clinical trial conducted in Botswana to address HIV NCD integration.

The InterCARE trial has some limitations which may limit generalizability. First, the clinics selected to participate in the trial have prior exposure to research principles. Second, although we distributed recruitment clinics geographically to cover most of the country, there are several remote areas where study set-up is not feasible with current resources.

In conclusion, the InterCARE trial has the potential to address the high burden of HTN in PLWH through integration into and leveraging well-established and successful HIV care program strategies in Botswana. The learning from InterCARE trial can inform integration of HTN and CVD risk management into HIV care more broadly in the region and globally.

## Trial status

Version 4. 27 November 2023. Recruitment began 31 January 2023 and completed 11 September 2023. Please note that the delay in submitting this protocol paper occurred due to the need to further adapt the implementation strategies from the pilot study (UG3) to the main trial (UH3).

## Data Availability

This study follows the NIH Public Access Policy, which ensures that the public has access to the published results of NIH funded research. All results will be made available from final peer-reviewed journal manuscripts via the digital archive PubMed Central upon acceptance for publication.

## Abbreviations

BP: Blood pressure
PWH: People with HIV
HIV: Human immunodeficiency virus
HTN: Hypertension
PWH and HTN: People with HIV and hypertension
CVD: Cardiovascular diseases
NCD: Non-communicable disease
ART: Antiretrovirals therapy
IDCC: Infectious Diseases Care Clinic
BCPP: Botswana HIV Combination Prevention Project
PIMS: Patient Information Management System
MoH: Ministry of Health
WHO: World Health Organization
UNAIDS: United Nations Program on HIV/AIDS
OPD: Outpatient department
HDFQ: Heart Disease Fact Questionnaire
REDCap: Research Electronic Data Capture
EHR: Electronic health records
CFIR: Consolidated Framework for Implementation Research
RE-AIM: Reach, Effectiveness, Adoption, Implementation, Maintenance
GLMM: Generalized linear mixed model

## References

[1] Bigna JJ, Noubiap JJ. Global burden of hypertension in people living with HIV. BMC Med. 2021;19(1):112. 33985500 10.1186/s12916-021-01981-y PMC8120709

[2] Mutemwa M, Peer N, de Villiers A, Mukasa B, Matsha TE, Mills EJ, et al. Prevalence, detection, treatment, and control of hypertension in human immunodeficiency virus (HIV)-infected patients attending HIV clinics in the Western Cape Province, South Africa. Medicine (Baltimore). 2018;97(35):e12121. 30170445 10.1097/MD.0000000000012121 PMC6392528

[3] Gaolathe T, Wirth KE, Holme MP, Makhema J, Moyo S, Chakalisa U, et al. Botswana’s progress toward achieving the 2020 UNAIDS 90–90-90 antiretroviral therapy and virological suppression goals: a population-based survey. Lancet HIV. 2016;3(5):e221–30. 27126489 10.1016/S2352-3018(16)00037-0 PMC5146754

[4] Muddu M, Tusubira AK, Sharma SK, Akiteng AR, Ssinabulya I, Schwartz JI. Integrated hypertension and HIV care cascades in an HIV treatment program in Eastern Uganda: a retrospective cohort study. J Acquir Immune Defic Syndr. 2019;81(5):552–61. 31045649 10.1097/QAI.0000000000002067 PMC6625912

[5] Masupe T, Tlhakanelo JT, Tiro MB, Motlhatlhedi K, Mamela A, Makwati O, et al. May Measurement Month 2018: an analysis of blood pressure screening results from Botswana. Eur Heart J Suppl. 2020;22(Suppl H):H23–5. 32884461 10.1093/eurheartj/suaa021 PMC7455262

[6] Mosepele M, Bennett K, Gaolathe T, Makhema JM, Mmalane M, Holme MP, et al. Prevalence and control of hypertension in a high HIV-prevalence setting, insights from a population based study in Botswana. Sci Rep. 2023;13(1):17814. 37857692 10.1038/s41598-023-44499-4 PMC10587125

[7] Ministry of Health & Wellness. Botswana Multi-Sectoral Strategy for the Prevention and Control of Non-Communicable Diseases. In: International Cancer Control Partnership (ICCP Portal). 2017. https://www.iccp-portal.org/system/files/plans/Botswana%20NCD%20Strategy%20Final.pdf. Accessed 13 June 2024.

[8] Ussery F, Bachanas P, Alwano MG, Lebelonyane R, Block L, Wirth K, et al. HIV incidence in Botswana rural communities with high antiretroviral treatment coverage: results from the Botswana Combination Prevention Project, 2013–2017. J Acquir Immune Defic Syndr. 2022;91(1):9–16. 35537094 10.1097/QAI.0000000000003017 PMC9388617

[9] Farahani M, Vable A, Lebelonyane R, Seipone K, Anderson M, Avalos A, et al. Outcomes of the Botswana national HIV/AIDS treatment programme from 2002 to 2010: a longitudinal analysis. Lancet Glob Health. 2014;2(1):e44–50. 25104635 10.1016/S2214-109X(13)70149-9

[10] Mishuris RG, Linder JA, Bates DW, Bitton A. Using electronic health record clinical decision support is associated with improved quality of care. Am J Manag Care. 2014;20(10):e445–52. 25414982

[11] Glasgow RE, Vogt TM, Boles SM. Evaluating the public health impact of health promotion interventions: the RE-AIM framework. Am J Public Health. 1999;89(9):1322–7. 10474547 10.2105/AJPH.89.9.1322 PMC1508772

[12] Bogart LM, Mosepele M, Phaladze N, Lekoko B, Klein DJ, MacCarthy S, et al. A social network analysis of HIV treatment partners and patient viral suppression in Botswana. J Acquir Immune Defic Syndr. 2018;78(2):183–92. 29465627 10.1097/QAI.0000000000001661 PMC5953800

[13] Conroy A, Leddy A, Johnson M, Ngubane T, van Rooyen H, Darbes L. ‘I told her this is your life’: relationship dynamics, partner support and adherence to antiretroviral therapy among South African couples. Cult Health Sex. 2017;19(11):1239–53. 28398134 10.1080/13691058.2017.1309460 PMC5626574

[14] National Medicine and Therapeutics Policy Advisory Committee (NMTPAC) and The AIDS and TB Directorate, Ministry of Health and Child Care. National-Drug-and-Therapeutics-Policy-Advisory-Committee. Guidelines for antiretroviral therapy for the prevention and treatment of HIV in Zimbabwe. 2013. https://www.childrenandaids.org/sites/default/files/2017-05/Zimbabwe_HIV-Guidelines-Dec2013.pdf. Accessed 13 June 2024.

[15] Katabira E, Kamya M, Kalyesubula I, Namale A. National antiretroviral treatment guidelines for adults, adolescents, and children. 2009. http://library.health.go.ug/sites/default/files/resources/National%20Antiretroviral%20treatment%20Guidelines%20for%20Adults%2CAdolescents%2C%20and%20children%202009.pdf. Accessed 13 June 2024.

[16] Ministry of Health, Lesotho. National guidelines on the use of antiretroviral therapy for HIV prevention and treatment. 2016. https://www.childrenandaids.org/sites/default/files/2017-04/Lesotho_ART-Guidelines_2016.pdf. Accessed 13 June 2024

[17] Bogart LM, Phaladze N, Kgotlaetsile K, Klein DJ, Goggin K, Mosepele M. Pilot test of Mopati, a multi-level adherence intervention for people living with HIV and their treatment partners in Botswana. Int J Behav Med. 2023. 10.1007/s12529-023-10233-7. 37957535 10.1007/s12529-023-10233-7 PMC11089073

[18] Billy Tsima VS, Oathokwa Nkomazana. Developing the Botswana primary care guidelines: an integrated, symptom based primary care guideline for the adult patient in a resource limited setting. J Multidiscip Healthc. 2016;9:347–54. 10.2147/JMDH.S112466 PMC4986912 27570457

[19] Ojji DB, Mayosi B, Francis V, Badri M, Cornelius V, Smythe W, et al. Comparison of dual therapies for lowering blood pressure in Black Africans. N Engl J Med. 2019;380(25):2429–39. 30883050 10.1056/NEJMoa1901113

[20] Chan AW, Tetzlaff JM, Altman DG, Laupacis A, Gøtzsche PC, Krleža-Jerić K, et al. SPIRIT 2013 statement: defining standard protocol items for clinical trials. Ann Intern Med. 2013;158(3):200–7. 23295957 10.7326/0003-4819-158-3-201302050-00583 PMC5114123

[21] Makhema J, Wirth KE, Pretorius Holme M, Gaolathe T, Mmalane M, Kadima E, et al. Universal testing, expanded treatment, and incidence of HIV infection in Botswana. N Engl J Med. 2019;381(3):230–42. 31314967 10.1056/NEJMoa1812281 PMC6800102

[22] Richard J. Hayes LHM. Cluster randomised trials. 2^nd^ ed. Chapman and Hall/CRC; 2017

[23] Damschroder LJ, Aron DC, Keith RE, Kirsh SR, Alexander JA, Lowery JC. Fostering implementation of health services research findings into practice: a consolidated framework for advancing implementation science. Implement Sci. 2009;4(1):50. 19664226 10.1186/1748-5908-4-50 PMC2736161

[24] Damschroder LJ, Reardon CM, Widerquist MAO, Lowery J. The updated Consolidated Framework for Implementation Research based on user feedback. Implement Sci. 2022;17(1):75. 36309746 10.1186/s13012-022-01245-0 PMC9617234

[25] Harris PA, Taylor R, Thielke R, Payne J, Gonzalez N, Conde JG. Research electronic data capture (REDCap)—a metadata-driven methodology and workflow process for providing translational research informatics support. J Biomed Inform. 2009;42(2):377–81. 18929686 10.1016/j.jbi.2008.08.010 PMC2700030

[26] Harris PA, Taylor R, Minor BL, Elliott V, Fernandez M, O’Neal L, et al. The REDCap consortium: building an international community of software platform partners. J Biomed Inform. 2019;95: 103208. 31078660 10.1016/j.jbi.2019.103208 PMC7254481

[27] Cohen J. A coefficient of agreement for nominal scales. Educ Psychol Measur. 1960;20(1):37–46. 10.1177/001316446002000104

[28] Palinkas LA, Horwitz SM, Green CA, Wisdom JP, Duan N, Hoagwood K. Purposeful sampling for qualitative data collection and analysis in mixed method implementation research. Adm Policy Ment Health. 2015;42(5):533–44. 24193818 10.1007/s10488-013-0528-y PMC4012002

[29] Government of Botswana, National AIDS & Health Promotion Agency. Botswana AIDS Impact Survey V (BAIS V) 2021. In: Unicef, Botswana. https://www.unicef.org/botswana/documents/botswana-hivaids-impact-survey-report-0. Accessed 13 June 2024.

